# Clinical and functional outcomes of arthroscopic autologous collagen‐induced chondrogenesis (ACIC) for osteochondral lesions of the talus: A retrospective case series

**DOI:** 10.1002/jeo2.70642

**Published:** 2026-01-27

**Authors:** Simone Daniel Gatti, Pierre‐Henri Vermorel, Jordi Vega, Jorge Batista, Matteo Guelfi

**Affiliations:** ^1^ School of Medicine and Surgery University of Milano‐Bicocca Monza Italy; ^2^ Orthopedic Department San Gerardo Hospital Monza Italy; ^3^ Inter‐University Laboratory of Human Movement Science University Lyon—University Jean‐Monnet Saint‐Étienne Saint‐Étienne France; ^4^ Department of Orthopaedic Surgery University Hospital Centre of Saint‐Étienne Saint‐Étienne France; ^5^ iMove Sports Barcelona Spain; ^6^ MIFAS by GRECMIP Merignac France; ^7^ Department of Arthroscopy Centro Artroscopico Jorge Batista SA Buenos Aires Argentina; ^8^ Sports Medicine Department Club Atletico Boca Juniors Buenos Aires Argentina; ^9^ Foot and Ankle Unit Casa di Cura Villa Montallegro Genoa Italy; ^10^ J Medical Turin Italy

**Keywords:** ACIC, ankle arthroscopy, cartilage repair, ChondroFiller, osteochondral lesions

## Abstract

**Purpose:**

The aim of this retrospective study was to assess the clinical outcomes for osteochondral lesions of the talus (OLT) treated with all arthroscopic autologous collagen‐induced chondrogenesis (ACIC) technique using an injectable collagen scaffold combined with microfractures.

**Methods:**

Between 2020 and 2023, 21 patients with Hepple Grade II–IV OLTs underwent arthroscopic ACIC technique using an injectable collagen scaffold combined with microfractures. Concomitant intra‐articular pathologies identified during arthroscopy were addressed simultaneously. Clinical outcomes, including Visual Analogue Scale (VAS), Foot Functional Index (FFI) and Foot and Ankle Ability Measure–Sports subscale (FAAM‐SS), were assessed preoperatively and at the latest follow‐up. Patient expectations, complications and return‐to‐activity times were also recorded at final follow‐up.

**Results:**

The mean follow‐up was 37.4 ± 20.7 (range, 13–68) months. The mean defect size was 76.6 ± 33.6 mm^2^ (range, 35–135 mm^2^). All ﻿clinical scores significantly improved compared with preoperative values (*p* < 0.05): VAS improved from 3.17 ± 1.82 (95% confidence interval [CI]: 2.34–4.00) to 0.27 ± 0.48 (95% CI 0.05–0.49), FFI from 40.90 ± 25.42 (95% CI: 29.33–52.47) to 4.02 ± 3.86 (95% CI: 2.26–5.78) and FAAM‐SS from 37.81 ± 28.51 (95% CI: 24.83–50.79) to 86.11 ± 13.75 (95% CI: 79.85–92.37). All patients reported that their expectations were met or exceeded regarding pain relief, functional recovery and return to daily activities. Associated intra‐articular pathologies were identified in all patients; in 17 patients (80.9%), a concomitant lateral ligament injury was observed and treated with arthroscopic repair. No major complications or revision surgeries occurred.

**Conclusions:**

Arthroscopic ACIC with an injectable atelocollagen scaffold was feasible and safe in this series of Hepple II–IV OLT, yielding improvements in patient‑reported outcomes at a mean 37.4‑month follow‑up. Due to design limitations and frequent concomitant ligament stabilization, these findings should be considered hypothesis‑generating rather than definitive evidence of cartilage regeneration.

**Level of Evidence:**

Level IV, retrospective case series.

**Abbreviations**
ACIC: autologous collagen‐induced chondrogenesis
AMIC: autologous matrix‐induced chondrogenesis
ATFL: anterior talofibular ligament
CFL: calcaneofibular ligament
CT: computed tomography
FAAM‐SS: Foot and Ankle Ability Measure—Sports Subscale
FFI: Foot Functional Index
IRB: institutional review board
LAS: lateral ankle sprains
LFTCL: lateral fibulotalocalcaneal ligament complex
MRI: magnetic resonance imaging
MSCs: mesenchymal stem cells
OLT: osteochondral lesions of the talus
ROM: range of motion
VAS: Visual Analogue Scale

## BACKGROUND

Osteochondral lesions of the talus (OLT) are a common complication following lateral ankle sprains (LAS), with a reported incidence of up to 6.5% [4]. They represent a significant clinical challenge due to the inherently limited regenerative potential of articular cartilage. If left untreated, these lesions can result in persistent pain, reduced joint function and eventually lead to degenerative joint changes and osteoarthritis [19].

Various surgical procedures have been developed to manage these lesions. Arthroscopic microfracture is an effective and widely used treatment due to its minimally invasive nature and favourable short‐term outcomes [3, 20]. Microfracture induces cartilage regeneration by stimulating the recruitment of mesenchymal stem cells (MSCs) from subchondral bone marrow. These MSCs possess significant chondrogenic potential and release cytokines that exhibit anti‐inflammatory properties, contributing to joint homoeostasis and fibrocartilaginous regeneration [23, 34]. To enhance the quality and durability of cartilage regeneration, recent techniques have combined microfracture with biological matrices designed to stabilize the blood clot and create an optimal environment for tissue regeneration. Autologous matrix‐induced chondrogenesis (AMIC) consists of the application of a solid collagen scaffold to secure the initial clot induced by microfractures, improving fibrocartilage quality [34]. Encouraging outcomes have been observed in mid‐term follow‐ups, particularly among athletes and physically active populations [9, 29].

The autologous collagen‐induced chondrogenesis (ACIC) technique involves the application of an injectable atelocollagen scaffold over the microfractured area to cover the defect and to retain the MSCs, favouring fibrocartilage formation [21]. After injection, the atelocollagen rapidly polymerizes within the defect, providing a structured microenvironment that supports cellular adhesion and chondrogenic differentiation, thereby stabilizing the marrow clot and facilitating repair. Preclinical evidence has shown that atelocollagen promotes MSC chondrogenesis in vitro [15], while a multicenter RCT in talar OLTs reported superior magnetic resonance imaging (MRI) repair tissue quality after microfracture with atelocollagen augmentation versus microfracture alone, with no statistically significant differences in clinical scores at 2 years [18]. The use of injectable scaffolds (ACIC) offers several theoretical advantages over solid membranes (AMIC), including simplified implantation and superior adaptability to the lesion site. Arthroscopy not only allows for minimally invasive cartilage repair but also permits comprehensive evaluation and management of concomitant intra‐articular pathologies through the same arthroscopic approaches [34].

Despite promising preclinical results and preliminary clinical reports, clinical evidence for ACIC in talar lesions remains limited, with heterogeneous case series and few comparative trials [15, 33, 34].

The aim of this study was to report clinical outcomes of patients treated for OLT with the arthroscopic ACIC technique. It was hypothesized that the all‐arthroscopic ACIC technique would yield favourable clinical outcomes, leading to improvements in pain, functional status and quality of life, particularly when concomitant ankle instability was simultaneously addressed.

## METHODS

This retrospective study was approved by the institutional review board of our institution (IRB number 2021‐09‐020).

Between 2020 and 2023, a total of 21 patients (14 males and 7 females) who underwent arthroscopic treatment for OLT using the ACIC technique were included. Inclusion criteria were skeletally mature patients (>15 years) with OLT Grade II–IV according to Hepple classification [13], lesion size smaller than 2 cm^2^ assessed intraoperatively and failed ≥6 months of conservative treatment and a minimum of 1‐year follow‐up. Exclusion criteria were advanced ankle arthritis, malalignment, tibial kissing lesions, subchondral cysts (Hepple Grade V), prior ankle surgery for OLT or systemic rheumatologic/neuromuscular disease, and patients who had not completed the 1‐year follow‐up.

**Figure 1:**
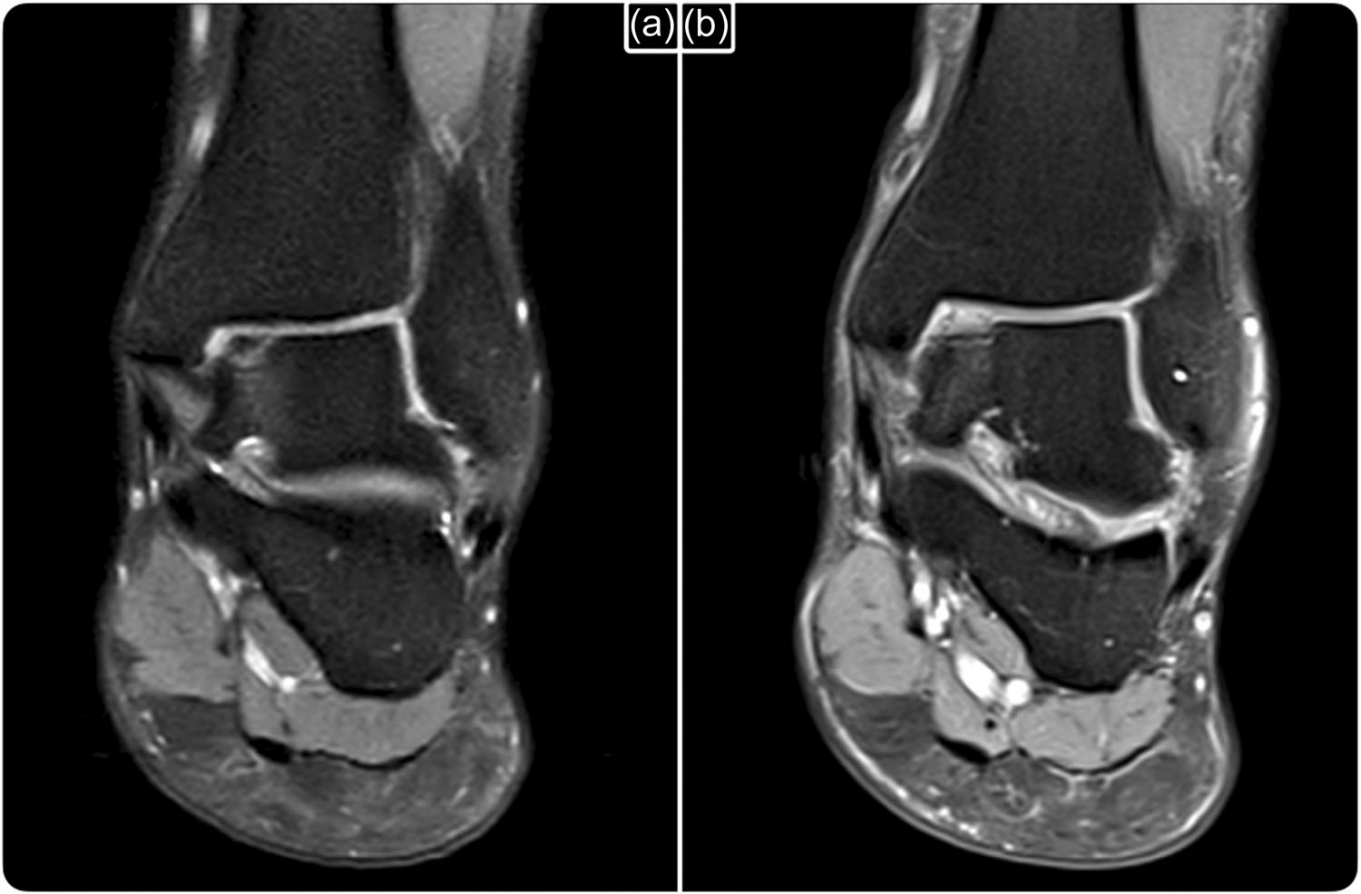
T2 MRI of a patient treated with the ACIC technique, preoperatively (a) and at 12 postoperatively (b), for an OLT located on the medial side. ACIC, autologous collagen‐induced chondrogenesis; MRI, magnetic resonance imaging; OLT, osteochondral lesions of the talus.

The preoperative diagnosis was based on patient history and a standardized clinical examination protocol, including gait assessment, inspection of ankle appearance, ligament stability testing and identification of tender points. Preoperative ankle MRI and computed tomography (CT) were obtained in all cases to classify the lesion according to the Hepple classification and implement the diagnosis (Figure 1).

Functional outcomes using the Foot Functional Index (FFI), Visual Analogue Scale (VAS) and Foot and Ankle Ability Measure–Sports subscale (FAAM‐SS) were assessed preoperatively and at the last follow‐up. At the last follow‐up, an institutional late postoperative follow‐up form was used to assess the timing (in weeks) of return to daily living, work and sporting activities after surgery. This form was also used to evaluate whether patient expectations were met regarding pain reduction, joint motion and strength, resumption of normal daily functions and return to sporting activities. Responses were categorized as ‘did not meet expectations’, ‘met expectations’ or ‘exceeded expectations’. This was an internal, non‑validated form used consistently across the study period. Postoperative complications were also recorded at final follow‐up.

### Surgical procedure

All the surgeries have been performed by a single orthopaedic surgeon specialized in foot and ankle surgery (M.G.). A no‐distraction and dorsiflexion arthroscopic technique was used in all cases. Patients underwent surgery under spinal anaesthesia, positioned supine with a thigh tourniquet. Cutaneous landmarks over the anterior and lateral aspects of the ankle were marked, and standard anteromedial and anterolateral portals were established. During diagnostic arthroscopy, intra‐articular pathologies such as synovitis, scar tissue or loose bodies were addressed before proceeding to the osteochondral lesion.

**Figure 2:**
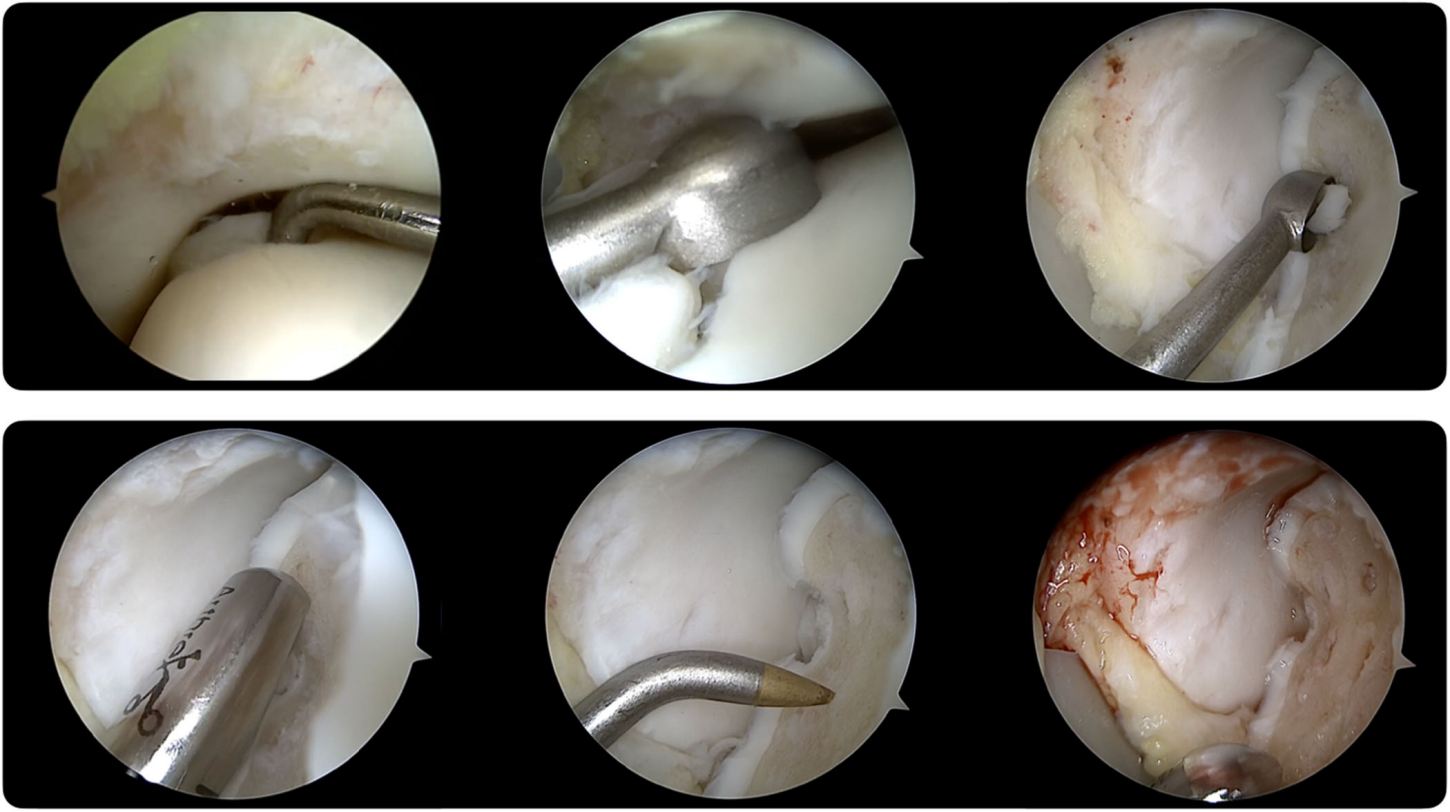
Step‐by‐step ACIC technique for OLT. With the camera placed in the medial portal, the cartilage lesion is identified, and then the portals are switched. The lesion is debrided with a curette and shaver until all damaged cartilage is removed, achieving well‐defined borders surrounded by healthy cartilage. Multiple microfractures are then performed. Finally, irrigation is stopped, and the joint is meticulously dried to optimize scaffold adhesion. ACIC, autologous collagen‐induced chondrogenesis; OLT, osteochondral lesions of the talus.

Osteochondral lesions were carefully evaluated, and their location was determined using the Raikin classification system [22]. Lesion size was measured intraoperatively. Unstable chondral flaps were meticulously debrided using motorized shavers until stable, well‐defined lesion borders surrounded by healthy cartilage were obtained. The damaged cartilage layer was thoroughly removed down to the subchondral bone. Multiple microfractures were then performed using a dedicated 1.8 mm chondral pick with a 4 mm depth, placed approximately 3–4 mm apart, to stimulate bone marrow access and induce subchondral bleeding (Figure 2).

**Figure 3:**
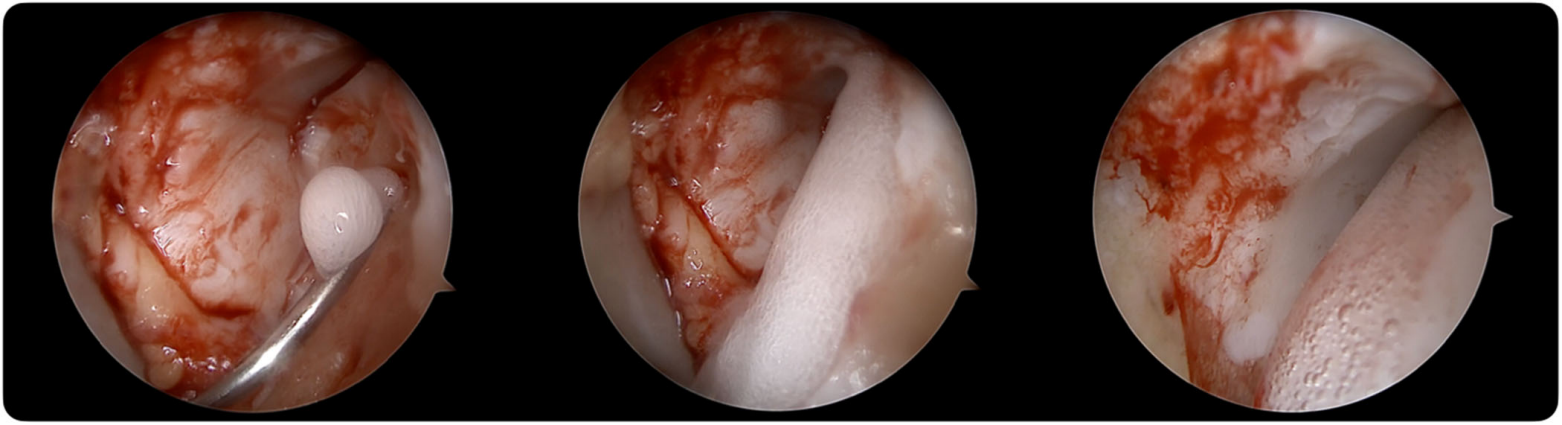
ChondroFiller® liquid, mixed with fibrin glue, is injected directly into the prepared defect under arthroscopic visualization. The matrix is applied gradually in thin layers until the defect is completely filled. After polymerization and solidification of the scaffold, irrigation is resumed, and the final cartilage repair and scaffold stability are assessed.

**Figure 4:**
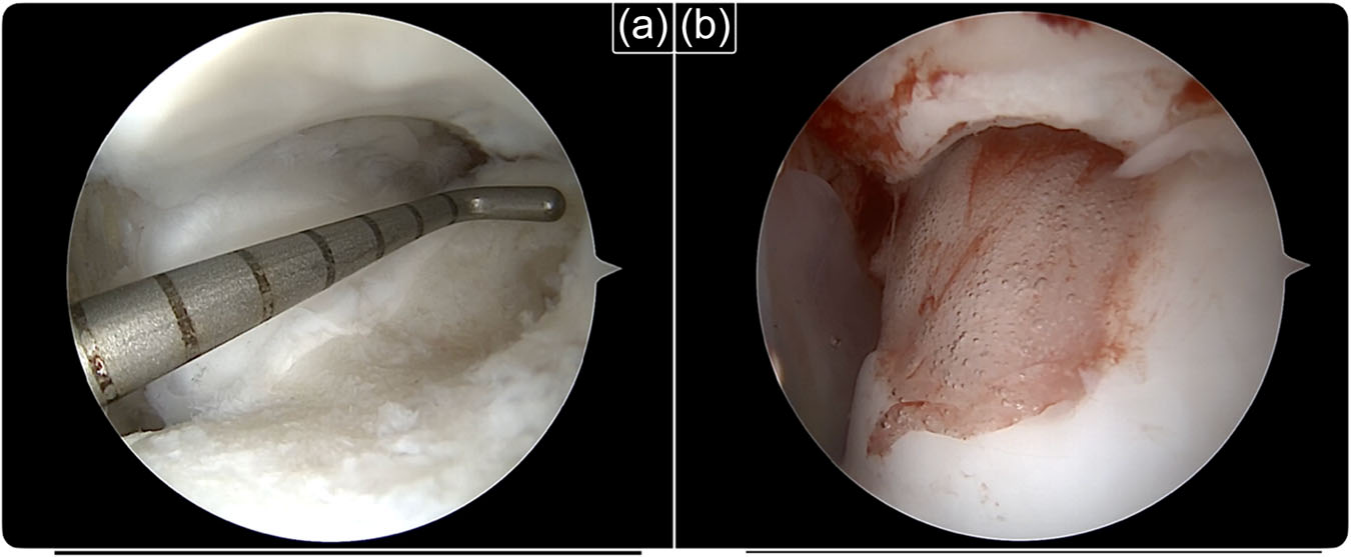
Arthroscopic view of a lateral OLT. On the left (a), the lesion size is measured intraoperatively after cartilage debridement; on the right (b), the final view shows the lesion completely filled with ChondroFiller® liquid (b). OLT, osteochondral lesions of the talus.

Subsequently, irrigation was stopped, and the joint was meticulously dried to optimize scaffold adhesion. Using a dual‐syringe system, 1 mL of ChondroFiller® liquid (Meidrix Biomedicals GmbH) was mixed in a 1:1 ratio with fibrin glue (Tisseel™; Baxter) and injected directly into the prepared defect under arthroscopic visualization. A curette was used to guide and evenly distribute the mixture, ensuring full contact with the lesion. The adjunctive use of fibrin glue was intended to enhance initial scaffold adhesion to the subchondral bone, reducing the risk of scaffold displacement and allowing resumption of irrigation if needed. The matrix was applied gradually in thin layers until the defect was filled flush with the surrounding cartilage. The joint was then maintained dry for 5 min approximately to allow polymerization and solidification of the initially transparent gel into a stable, whitish matrix (Figure 3). Scaffold stability was confirmed with gentle passive ankle motion under direct visualization (Figure 4).

In cases where medial or lateral ligamentous injury was identified during the procedure, repair was performed only after completion of the osteochondral repair. This sequencing was chosen to avoid stressing the ligament repair during cartilage treatment and to allow easier access to the posterior talar zones. Once the scaffold had polymerized and its adherence was confirmed, irrigation was resumed and the arthroscopy continued. All ligament repairs were performed arthroscopically using an all‐inside technique, as originally described in the literature [11, 12, 30, 31].

### Postoperative management

Postoperatively, all patients followed a standardized rehabilitation protocol tailored to the ACIC technique. The initial phase included a strict non‐weight‐bearing period of 3 weeks, during which the ankle was immobilized in a walker boot. This was followed by a gradual progression to full weight‐bearing as tolerated, with continued use of the walker boot and crutches until satisfactory load tolerance was achieved. Open‐chain exercises for restoring range of motion (ROM) and joint mobility were initiated immediately after surgery in patients who did not undergo any ligamentous repair. In contrast, for patients who underwent concomitant ligament repair, ROM exercises were postponed and typically initiated between the second and third postoperative week, depending on the stability of the ligament repair and clinical judgement. This protocol was designed to protect the scaffold during the initial healing phase while promoting early joint mobilization, crucial for preserving ankle function.

### Statistical analysis

Continuous variables, including the Visual Analogue Scale (VAS), Foot Function Index (FFI) and Foot and Ankle Ability Measure—Sports Subscale (FAAM‐SS), were collected preoperatively and at 12‐month follow‐up. The distribution of paired differences was assessed for normality using the Shapiro–Wilk test. For normally distributed data, comparisons between pre‐ and postoperative values were made using paired *t* tests. When data were not normally distributed, the Wilcoxon signed‐rank test was employed. A *p* value less than 0.05 was considered statistically significant.

A post hoc power analysis for the primary outcomes, based on a paired‐samples design, yielded power estimates >0.99 for all comparisons, confirming that the study was adequately powered to detect the observed differences.

Statistical analyses were conducted using SPSS (IBM Corp.).

## RESULTS

### Patient demographics

The study cohort comprised 21 patients who underwent arthroscopic ACIC treatment for OLT. The mean age of the population was 35.5 ± 15.9 years. The left ankle was affected in 11 of the 21 cases. The mean follow‐up was 37.4 ± 20.7 months (range, 13–68 months).

### Lesion characteristics

The lesions were classified as Grade II–IV according to the Hepple classification system [13]. The mean defect size was 76.6 ± 33.6 mm^2^ (range, 35–135 mm^2^). Five patients presented with a lesion size greater than 100 mm^2^. The mean depth of the lesions, calculated from arthroscopic evaluation, was 5.0 ± 0.9 mm. Using the Raikin system [22], lesion locations were distributed as follows: 17 lesions were medial (8 patients in Zones 4–7, 4 patients in Zone 4, 3 patients in Zone 7 and 2 patients in Zones 1–4–7) and 4 were lateral (3 patients in Zone 6, 1 patient in Zones 3–6). Posterior involvement (posterior zones on the Raikin grid) was present in 13/21 patients (61.9%): 3 had isolated posteromedial lesions (zone 7), and 10 had lesions extending posteriorly (8 across Zones 4–7 and 2 across Zones 1–4–7). Patient demographics and lesion characteristics are summarized in Table 1.

**Table 1:** Patient demographics and osteochondral lesion characteristics.

Demographics	
No. of patients	21
Sex (male/female)	14/7
Mean age (years)	35.5 ± 15.9
Mean follow‐up (months)	37.4 ± 20.7
Side (left/right)	11/10
Lesion location (medial/lateral)	17/4
Mean defect depth (mm)	5.0 ± 0.9
Mean defect size (mm^2^)	76.6 ± 33.6

All patients presented with associated intra‐articular pathologies. Concomitant ligament injuries were observed in 17 patients (80.9%), including 13 (61.9%) with isolated anterior talofibular ligament (ATFL) injury and 4 (19%) with combined ATFL and deltoid ligament injuries. The lateral collateral ligament injury involved only the superior fascicle of the ATFL in 10 patients, whereas in 7 patients it extended to the ATFL component of the lateral fibulotalocalcaneal ligament complex (LFTCL). No injuries of the CFL component of the LFTCL complex were observed. Only 4 patients (18.1%) had OLT without any associated ankle ligament injury. Additionally, 3 patients (14.3%) presented intra‐articular loose bodies, and 3 (14.3%) had significant synovitis. Associated pathologies are summarized in Table 2.

**Table 2:** Associated pathologies.

Associated pathology	Patients (*n*, %)
Type of ligament injury	
Isolated ATFL	13 (61.9)
Combined ATFL and deltoid injury	4 (19)
Intra‐articular loose bodies	3 (14.3)
Synovitis	3 (14.3)

Abbreviation: ATFL, anterior talofibular ligament.

### Clinical outcomes

Significant improvements were observed in all clinical scores at final follow‐up compared with preoperative values (*p* < 0.05). VAS improved from 3.17 ± 1.82 (95% CI: 2.34–4.00) to 0.27 ± 0.48 (95% CI: 0.05–0.49) (*p* = 0.008), FFI from 40.90 ± 25.42 (95% CI: 29.33–52.47) to 4.02 ± 3.86 (95% CI: 2.26–5.78) (*p* = 0.002) and FAAM‐SS from 37.81 ± 28.51 (95% CI: 24.83–50.79) to 86.11 ± 13.75 (95% CI: 79.85–92.37) (*p* = 0.002).

**Figure 5:**
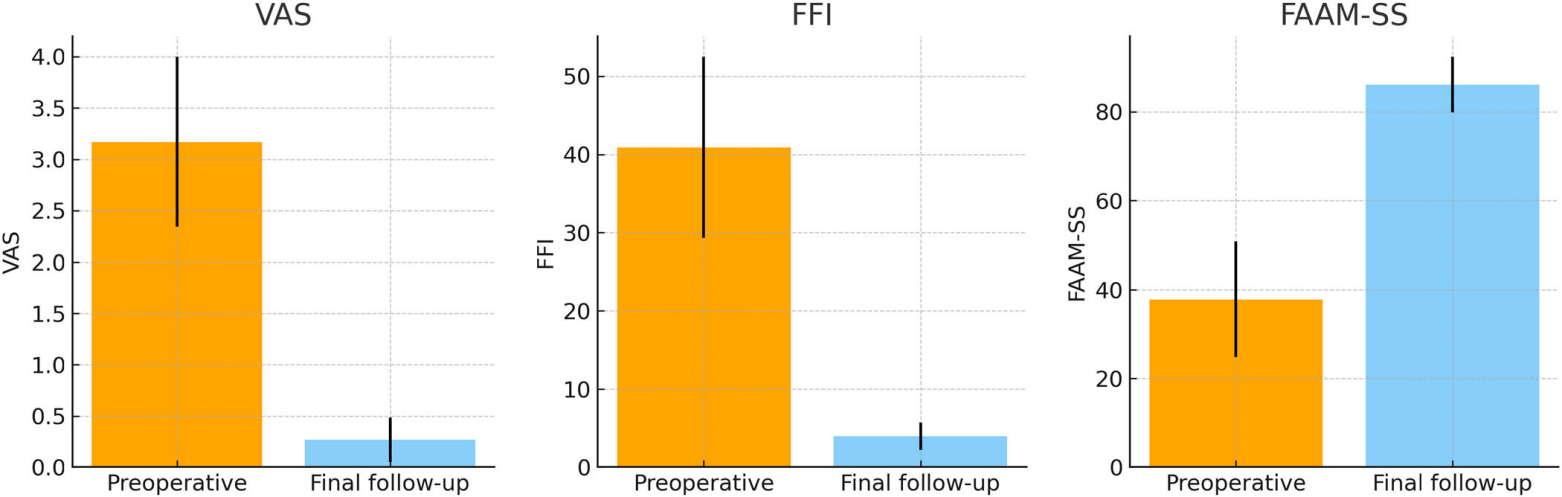
Patient‐reported outcome measures (PROMs)—VAS, FFI and FAAM‐SS at preoperative baseline and final follow‐up. Bars show means with 95% CI; orange = preoperative, blue = final follow‐up; *n* = 21. Pre–post comparisons: VAS *p* = 0.008; FFI *p* = 0.002; FAAM‐SS *p* = 0.002. *Note*: Lower values indicate better outcomes for VAS and FFI, whereas higher values indicate better outcomes for FAAM‐SS. CI, confidence interval; FAAM‐SS, Foot and Ankle Ability Measure—Sports Subscale; FFI, Foot Functional Index; VAS, Visual Analogue Scale.

Pre‐to‐post improvements for all patient‐reported outcomes are illustrated in Figure 5, which reports means with 95% CIs (*n* = 21).

### Complications and return to activity

No major complications or revision surgeries were reported within the follow‐up period.

Regarding the return to daily living activities, 13 patients (61.9%) resumed activities between 6 and 12 weeks, while 8 patients (38.1%) returned between 18 and 24 weeks. Concerning return to work, 16 patients (76.2%) returned between 6 and 12 weeks, and 5 patients (23.8%) returned between 18 and 24 weeks. As for return to sports activities, 2 patients (9.5%) returned between 12 and 18 weeks, 4 patients (19.0%) between 18 and 24 weeks, and 9 patients (42.8%) required more than 24 weeks. Six patients did not practice any sport (Table 3).

**Table 3:** Return to daily activities, sports activities and work after surgery.

Activity	Time interval (weeks)	Patients (*n*, %)
Return to daily activities	6–12	13 (61.9)
	18–24	8 (38.1)
	>24	0 (0)
Return to work	6–12	16 (76.2)
	18–24	5 (23.8)
	>24	0 (0)
Return to sports	12–18	2 (9.5)
	18–24	4 (19.0)
	>24	9 (42.8)
	Did not practice sports	6 (28.6)

At the final follow‐up, all 21 patients responded positively to the expectations concerning pain reduction, with 11 patients (52.4%) reporting that the treatment met their expectations and 10 patients (47.6%) stating it exceeded their expectations. In terms of improvement in motion and joint strength, 15 patients (71.4%) reported meeting their expectations, while 6 (28.6%) reported exceeding their expectations. Regarding the resumption of normal daily living functions, all 21 (100%) responding patients indicated that the treatment exceeded their expectations. Finally, among those involved in sports activities, 15 patients (71.4%) reported that their expectations were met; for 6 patients (28.6%), this question was not applicable as they did not practice any sport.

## DISCUSSION

The most important finding of this study is that the arthroscopic ACIC technique using an injectable collagen scaffold is effective for the treatment of OLT, providing significant clinical outcomes with significant pain reduction and functional improvement. The frequent association of OLT with intra‐articular pathologies, particularly ligamentous injuries, highlights the value of arthroscopy, which allows minimally invasive cartilage repair along with simultaneous management of associated lesions.

These favourable clinical outcomes align with existing literature supporting scaffold‐enhanced techniques, which aim to stabilize the blood clot formed by microfractures and potentially improve cartilage regeneration [28]. Previous studies have reported durable clinical improvements using microfracture alone, raising questions regarding the necessity and effectiveness of additional scaffolds [1, 3]. Our postoperative scores fall within the range commonly reported for both microfracture/BMS and AMIC at short‐ to mid‐term follow‐up, although direct numeric equivalence is limited by scale heterogeneity and differences in lesion mix and concomitant procedures. However, several theoretical advantages justify the ongoing exploration of collagen‐based matrices, including improved clot containment and potentially more favourable mechanical properties of the initial repair tissue [14]. ACIC techniques, such as ChondroFiller® liquid, provide a structured environment that potentially facilitates improved cellular colonization, differentiation and cartilage regeneration [18]. Lesion characteristics, particularly lesion size, may influence the decision to employ scaffold augmentation. Literature suggests that larger lesions (>1 cm^2^) may particularly benefit from collagen‐based augmentation due to difficulties maintaining clot stability and containment, highlighting a subgroup where the addition of scaffolds could be especially advantageous [5, 6, 8, 10].

In the present study, a majority of patients resumed daily living activities (61.9%) and returned to work (76.2%) within 6–12 weeks postoperatively. Return‐to‐sport intervals showed greater variability (9.5% returned between 12 and 18 weeks, 19% between 18 and 24 weeks, and 42.8% took longer than 24 weeks), likely reflecting individual patient factors, lesion characteristics and the nature of the associated ligamentous procedures. These findings are consistent with existing reports, which typically indicate high return‐to‐sport rates (86%–97%) and mean times ranging from 13 to 26 weeks after OLT treatment, although not always at pre‐injury activity level [17, 24].

The safety profile of the ACIC technique observed in the present study did not reveal any additional risks compared with standard arthroscopic microfracture procedures. Its feasibility as a fully arthroscopic procedure represents a significant advantage, particularly given the limited surgical accessibility of the ankle joint [34]. Furthermore, in contrast to open cartilage restoration techniques such as autologous chondrocyte implantation (ACI) or osteochondral autologous transplantation (OATS), ACIC provides the advantages of reduced donor‐site morbidity, simplified procedural logistics and immediate availability, which may improve patient acceptance and overall satisfaction [26]. Compared with the AMIC technique, ACIC may facilitate treatment of more posterior lesions owing to the injectable liquid scaffold formulation [2]. In addition, it eliminates the need for pre‐modelling and precise lesion measurement, which can be technically challenging in anatomically complex regions. In clinical practice, these features render the ACIC procedure technically more straightforward.

An important finding of the present study was the high prevalence of ligament injuries in patients with OLT, observed in 80.9% of cases. Concurrent recognition and management of these injuries are crucial, as untreated ankle instability may compromise clinical outcomes [25, 32, 36]. It must be recognized that the high rate of concurrent ligament repair raises the possibility that the clinical improvements observed in VAS, FFI and FAAM‐SS scores are primarily driven by ligament stabilization rather than the ACIC technique alone. Without a control group evaluating isolated ACIC or a subgroup analysis comparing patients with and without ligament repair, the independent effect of the injectable collagen scaffold cannot be definitively determined. However, unrecognized ankle instability may initiate a domino effect, leading to recurrent ankle sprains and progressive worsening of mechanical instability, thereby predisposing the joint to irreversible intra‐articular damage [7]. These results underscore the added value of ankle arthroscopy for accurate assessment of both intra‐articular pathology and ligamentous integrity, enabling comprehensive treatment of OLT and associated instability.

This study has several limitations. Its retrospective single‐surgeon design carries inherent risks, including potential selection bias and limited extrapolability. Nevertheless, this design allowed evaluation of real‐world clinical outcomes at the last follow‐up in a relatively large cohort of patients with OLT, providing valuable insights into current treatment strategies. While a larger cohort would have improved internal and external validity, post hoc power analysis confirmed that the study was adequately powered. The absence of a control group also limits interpretability, and a randomized controlled trial would be necessary to strengthen these findings. Crucially, the reliance solely on subjective outcome measures (VAS, FFI, FAAM‐SS) and the absence of postoperative objective imaging (MRI/CT) or second‐look arthroscopy prevent any definitive conclusion regarding cartilage regeneration or the quality of the repair tissue. Another limitation is the lack of intermediate follow‐up assessments, which could have provided information on the dynamics of functional recovery. Previous studies have reported variable trends over time, with Kim et al. showing continued improvement between 1 and 2 years [16], Usuelli et al. reporting a similar pattern in patients treated with AMIC [27], and Wei et al. observing no significant change over the same period [35]. Further prospective studies are warranted to better characterize the temporal evolution of functional outcomes. Finally, the type of sport practiced may influence the time to return to sport, as impact or pivoting sports are more demanding on the ankle and therefore may require a longer recovery compared with non‐impact sports.

The clinical relevance of this study is that the arthroscopic ACIC using an injectable collagen‐based scaffold can be feasibly, safely and effectively used for the treatment of OLT, thereby potentially reducing the morbidity associated with more invasive techniques. Furthermore, it is emphasized that concurrent ankle instability should be carefully evaluated and addressed, with ligamentous repair combined with cartilage repair to provide a biomechanically optimized environment for healing.

## CONCLUSIONS

Arthroscopic ACIC with injectable collagen scaffold provides significant improvements in clinical and functional scores in the treatment of Hepple Grade II–IV OLT. This technique represents a feasible and safe approach, combining minimally invasive cartilage repair with comprehensive arthroscopic management of concomitant intra‐articular pathologies, including associated ligamentous injuries. Due to design limitations and frequent concomitant ligament stabilization, these findings should be considered hypothesis‑generating rather than definitive evidence of cartilage regeneration.

## AUTHOR CONTRIBUTIONS

**Simone Daniel Gatti**: Conceptualization; methodology; writing. **Pierre‐Henri Vermorel**: Conceptualization; methodology; data analysis. **Jordi Vega**: Writing; editing. **Jorge Batista**: Methodology; editing. **Matteo Guelfi**: Conceptualization; methodology; writing.

## CONFLICT OF INTEREST STATEMENT

The authors declare no conflicts of interest.

## ETHICS STATEMENT

Ethics permission was obtained as disclosed in the methodology of the manuscript (IRB number 2021‐09‐020). No informed consent was needed in this study because this is a retrospective chart review of standard care procedures of de‐identified patients.

## Data Availability

All data generated or analysed during this study are included in this published article.
